# Botulinum Toxin: A Comprehensive Review of Its Molecular Architecture and Mechanistic Action

**DOI:** 10.3390/ijms26020777

**Published:** 2025-01-17

**Authors:** Raj Kumar, Bal Ram Singh

**Affiliations:** Botulinum Research Center, Institute of Advanced Sciences, Dartmouth, MA 02747, USA; bsingh@inads.org

**Keywords:** botulinum neurotoxin, Clostridiaceae, Clostridium family, *Bacillus* sp., E-cadherin, BoNT/NTNHA-like component A, SNARE proteins, TNF, SNAP-25, SNAP-23

## Abstract

Botulinum toxin (BoNT), the most potent substance known to humans, likely evolved not to kill but to serve other biological purposes. While its use in cosmetic applications is well known, its medical utility has become increasingly significant due to the intricacies of its structure and function. The toxin’s structural complexity enables it to target specific cellular processes with remarkable precision, making it an invaluable tool in both basic and applied biomedical research. BoNT’s potency stems from its unique structural features, which include domains responsible for receptor recognition, membrane binding, internalization, and enzymatic cleavage. This division of labor within the toxin’s structure allows it to specifically recognize and interact with synaptic proteins, leading to precise cleavage at targeted sites within neurons. The toxin’s mechanism of action involves a multi-step process: recognition, binding, and catalysis, ultimately blocking neurotransmitter release by cleaving proteins like SNAP-25, VAMP, and syntaxin. This disruption in synaptic vesicle fusion causes paralysis, typically in peripheral neurons. However, emerging evidence suggests that BoNT also affects the central nervous system (CNS), influencing presynaptic functions and distant neuronal systems. The evolutionary history of BoNT reveals that its neurotoxic properties likely provided a selective advantage in certain ecological contexts. Interestingly, the very features that make BoNT a potent toxin also enable its therapeutic applications, offering precision in treating neurological disorders like dystonia, spasticity, and chronic pain. In this review, we highlight the toxin’s structural, functional, and evolutionary aspects, explore its clinical uses, and identify key research gaps, such as BoNT’s central effects and its long-term cellular impact. A clear understanding of these aspects could facilitate the representation of BoNT as a unique scientific paradigm for studying neuronal processes and developing targeted therapeutic strategies.

## 1. Introduction

Clostridium, a genus of Gram-positive bacteria from the *Clostridiaceae* family, has been evolving for over 2.6 billion years. Among the many molecules produced by these bacteria, botulinum toxin (BoNT or BTX) stands out as the most potent neurotoxin known to humanity. The toxin is primarily produced by *Clostridium botulinum*, but other species such as *Clostridium butyricum*, *Clostridium baratii*, and *Clostridium argentinensis* are also capable of producing various forms of this toxin [1]. Botulinum toxin is found ubiquitously in the environment, often in the form of spores, and is associated with several clinical syndromes, most notably botulism. The condition can manifest after the ingestion of contaminated food, wound infection, or colonization of the gastrointestinal tract in infants, a serious and potentially fatal condition.

As a bioterrorism agent, botulinum toxin is classified as a Category A agent by the Centers for Disease Control and Prevention (CDC) due to its potency and potential for misuse. Despite the common pathological outcome of botulism across various serotypes, these exhibit relatively low sequence identity. For example, the amino acid sequence diversity among the different serotypes ranges from 37.2% to 69.6%, while the nucleotide sequence diversity ranges from 24.5% to 44.7% [2,3]. These genetic differences contribute to variations in the clinical manifestations and the clinical management of botulism, but the basic mechanism of action—disruption of neuromuscular transmission—remains consistent across serotypes.

Botulinum toxin’s ability to block the release of acetylcholine at the neuromuscular junction has made it an invaluable tool in treating a wide range of medical conditions. Therapeutically, it is used for chronic migraines, spasticity, an overactive bladder, excessive sweating, and more. In the field of esthetics, it is widely known for its wrinkle-reducing effects. As botulinum toxin’s medical applications continue to expand, so too does the need for a deeper understanding of its complex biology, potential long-term effects, and diverse clinical applications. While the clinical success of botulinum toxin has been impressive, many critical questions remain. For example, how does the toxin interact with the body over long periods? What are the long-term risks of repeated use, particularly in cosmetic settings? How can we enhance the therapeutic efficacy of botulinum toxin while minimizing its potential side effects?

To answer these questions, it is essential to examine the various facets of botulinum toxin, starting with its synthesis in Clostridium botulinum bacteria and continuing through to its molecular mechanisms in target cells. Despite extensive research into its biological properties, many mysteries surrounding botulinum toxin’s molecular structure, pathogenesis, and precise interactions with host cells remain unresolved.

The therapeutic potential of botulinum toxin is continually growing, as researchers uncover new uses and novel delivery mechanisms to improve human health. The discovery of previously unknown therapeutic applications has added to botulinum toxin’s clinical value, but a deeper understanding of its molecular biology is critical for ensuring its safe and effective use in diverse therapeutic settings.

This review aims to shed light on several key areas that remain poorly understood about botulinum toxin. Specifically, we will explore the following aspects:

- *Genomic Organization and Horizontal Gene Transfer:* Botulinum toxin’s synthesis is tightly regulated by the genes within Clostridium botulinum. One intriguing question is the role of horizontal gene transfer in shaping the genomic organization of these bacteria. How do mobile genetic elements, such as plasmids and bacteriophages, influence the diversity of botulinum toxin serotypes, and what implications might this have for its pathogenicity?
- *Structural Composition and Mechanism of Action:* While the general mechanism of botulinum toxin action is well understood—namely, its ability to cleave proteins involved in neurotransmitter release—many details about its structure remain under active investigation. How do variations in the structural components of the toxin influence its potency and specificity for different target cells? Furthermore, what molecular adaptations allow it to bind with such high specificity to neuronal receptors?
- *Interactions with Intestinal Epithelial Cells:* In addition to its well-known action on neurons, there is increasing interest in botulinum toxin’s interactions with intestinal epithelial cells, especially in cases of foodborne botulism. How does the toxin cross the intestinal epithelium to reach its target tissues? Does botulinum toxin utilize specific transport mechanisms for absorption, and how do these processes differ in infant botulism, where gastrointestinal colonization is the primary route of infection?
- *Potential Effects on the Central Nervous System (CNS):* While botulinum toxin primarily affects peripheral neurons, recent research has suggested that it may also influence the central nervous system. Does botulinum toxin cross the blood–brain barrier, and if so, what are the potential implications for neurological disorders and the development of new therapeutic strategies? Can botulinum toxin have direct effects on central nervous system disorders, and what would be its therapeutic potential in this regard?
- *Interactions with Associated Proteins and Pathogenesis:* Botulinum toxin interacts with a variety of host proteins during its entry into cells and its subsequent action. Understanding the full scope of these interactions is crucial for understanding its pathogenesis. Are there any novel protein interactions that could reveal new targets for therapeutic intervention, and could these interactions extend beyond neurons to other cell types?

Through this review, we aim to address these critical questions, providing a comprehensive understanding of the intricate biology of botulinum toxin. By expanding our knowledge of its genomic structure, cellular interactions, and broader therapeutic potential, we hope to contribute to its more targeted and effective use in both clinical and esthetic contexts. We will also discuss how emerging research may shape the future of botulinum-toxin-based therapies, exploring its evolving role in modern medicine and the potential for novel applications in both human health and disease.

## 2. Organizational Intricacies

Genetic sequencing has opened up new avenues for understanding the underlying diversity within the botulinum toxin (BoNT) family. Recent studies have identified two distinct classes of gene clusters across different BoNT serotypes, shedding light on their genetic variation and pathogenic mechanisms [3]. One type of cluster contains three hemagglutinin genes (*ha+/orf-*; *contains HA33*, *HA17*, and *HA70*; *ha70-ha17-ha33-botR-ntnha-bont*), where HA or *ha* refers to hemagglutinin activity, whereas the other type of cluster encodes four genes of unknown functions (*orf+/ha-*, *contains OrfX1*, *OrfX2*, *OrfX3*, and *P47; orfX3-orfX2-orfX1-botR-p47-ntnha-bont*) [3]. The genetic diversity observed among botulinum toxin (BoNT) serotypes is largely attributed to underlying genetic events such as insertions, deletions, and recombination, which may result from horizontal gene transfer (HGT) between bacterial strains. This gene transfer plays a significant role in creating the diversity within the BoNT family. Advances in genetic sequencing have revealed that certain *Clostridium botulinum* strains harbor multiple toxin genes within their genomes [4]. Interestingly, these strains typically express only one of the toxin genes at a time, while the other toxin genes remain silent. This observation suggests the presence of complex regulatory mechanisms that control toxin gene expression.

Further complexity arises from the occurrence of chimeric toxins, such as BoNT D/C, C/D, or A/F. These chimeric toxins are the result of past recombination events between different BoNT serotypes, leading to the creation of hybrid toxins with unique biological activities. Such genetic events contribute to the variability of botulinum toxin, influencing factors like its potency, host specificity, and clinical manifestations.

In addition to genetic diversity in toxin types, *C. botulinum* strains exhibit differences in the mode of toxin activation. Some serotypes, such as type B and type F, exist in two distinct forms: proteolytic and nonproteolytic. Proteolytic toxins undergo endogenous cleavage of the progenitor polypeptide into its active form, while nonproteolytic toxins require external cleavage to be activated. These differences are not intrinsic to the toxin itself but are likely influenced by the bacterial species or strain producing the toxin. Genetic sequencing has shown that these variations in activation mechanisms are tied to the metabolic characteristics of the bacterial strains, particularly the presence or absence of proteolytic enzymes necessary for endogenous cleavage.

Despite these physiological differences, the target of all BoNT serotypes remains consistent: the intracellular SNARE (soluble N-ethylmaleimide-sensitive factor attachment protein receptor) proteins. These proteins are involved in the exocytosis of acetylcholine, a neurotransmitter essential for muscle contraction and communication between neurons (see Table 1). The binding and cleavage of SNARE proteins by BoNT leads to the blockade of neurotransmitter release, resulting in the paralyzing characteristic of botulism.

The genetic complexity of BoNT is a remarkable feat of evolution, as *Clostridium botulinum* strains exhibit diverse chromosomal locations of their toxin genes, variations in their target species, and selective intracellular specificity. This diversity also extends to the abundance of subtypes within each serotype, which further complicates the classification and clinical management of botulism [3]. The G + C content of BoNT genes is 26.3%, which closely resembles that of *C. botulinum* genomes (28.2%) [5]. This similarity in base composition supports the hypothesis that these toxins have evolved from a common ancestral strain, with ongoing genetic exchange contributing to their diversity.

Gene transfer events among *C. botulinum* strains can lead to the acquisition of multiple neurotoxin genes, such as A(B), A(F), or B(F), resulting in strains that produce a broader range of toxin types. Such gene transfer events are often accompanied by additional genetic changes, such as mutations, deletions, and recombination, which can involve mobile DNA elements or the mobilization of large plasmids [6]. While gene transfer from eukaryotes or multicellular organisms to bacteria is a rare phenomenon, the specificity of BoNT’s action on eukaryotic cells raises the possibility of horizontal gene transfer (HGT) between *Clostridium* species and human or other multicellular organisms. This hypothesis, although speculative, cannot be entirely ruled out, given the toxin’s highly specialized targeting mechanisms.

## 3. Structural Composition

**Figure 1:**
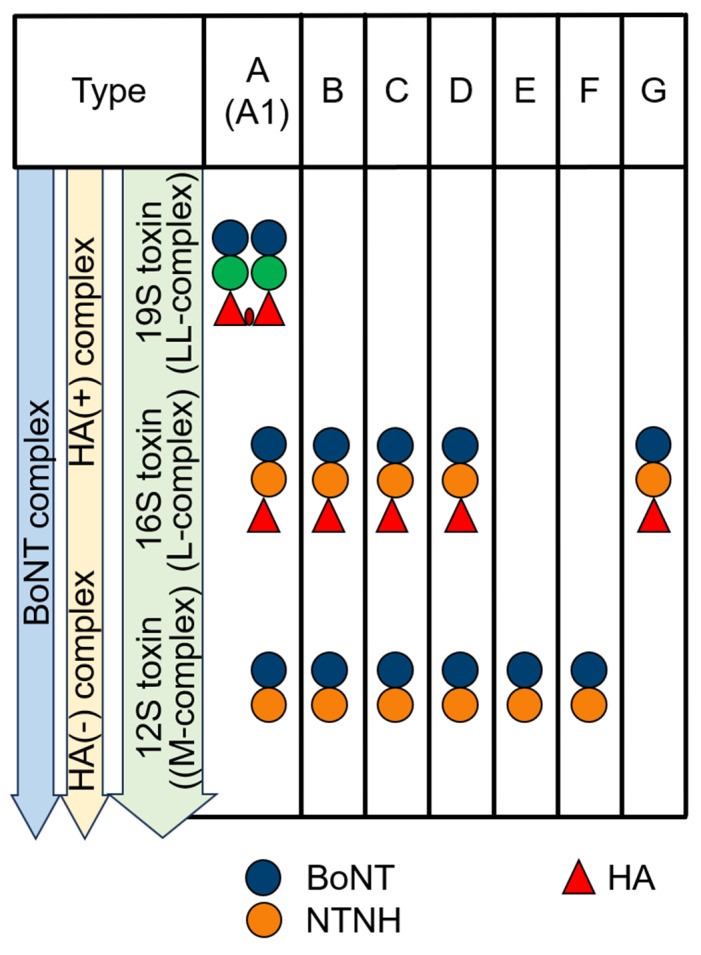
Different forms of botulinum complexes.

**Figure 2:**
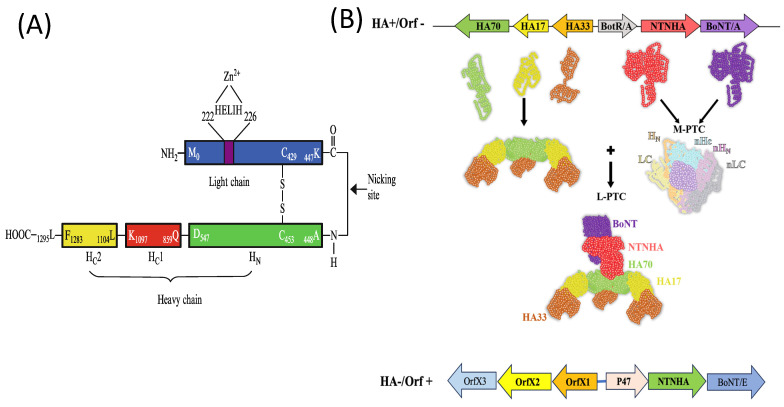
Structure and Components of the Botulinum Toxin A Complex. (**A**) The domains of Botulinum toxin A (BoNT/A) are illustrated. The 150 kDa BoNT/A consists of a light chain (LC), heavy chain (HC), and the translocation domain. The active site is indicated, highlighting the critical zinc-binding site and the hydrolysis mechanism essential for the neurotoxin’s proteolytic activity. (**B**) Schematic representation of Botulinum toxin complex A. This includes the BoNT/A protein and its associated neurotoxin-associated proteins (NAPs). The figure distinguishes between HA+/Orf- and HA-/Orf+ gene clusters, highlighting their distinct genetic organization and the resulting functional properties of the toxin complex. The role of HA proteins in the toxin’s stability and OrfX’s potential regulatory functions are also depicted.

As previously mentioned, two distinct types of botulinum toxin (BoNT) complexes exist: HA-positive (HA^+^) and OrfX-positive (OrfX^+^) BoNT complexes. Among the *Clostridium botulinum* serotypes, only Type A produces three different forms of BoNT complexes: the M complex (12S; ~300 kDa), the L complex (16S; ~500–600 kDa), and the LL complex (large/large; a dimer of two 16S complexes forming a 19S complex; ~900 kDa) (Figure 1) [3,7]. Other *C. botulinum* serotypes typically produce either the M (12S) or L (16S) complexes. The size of isolated botulinum toxin is approximately 150 kDa, consisting of two primary domains: the heavy chain (HC; ~100 kDa) and the light chain (LC; ~50 kDa) (Figure 1A). The heavy chain is further divided into three subdomains: Hc1 (or HCN domain; ~25 kDa), Hc2 (or HCC domain; ~25 kDa), and HN (the N-terminal domain; ~50 kDa) (Figure 2A). In the HA^+^ botulinum toxin complexes, the components include BoNT itself, NTNHA (Non-toxin Non-hemagglutinin Activity, which serves as the neurotoxin-binding protein), and hemagglutinin proteins (HA70, HA17, HA33). Together, these proteins are referred to as neurotoxin-associated proteins (NAPs) [7]. The ratio of these components varies across different studies, such as 1:1:2:2:3 [8], 1:1:3-5:5-6:8-9 [9], or 1:1:3:3:3 [10], with some studies specifically investigating BoNT/A L complexes [11]. In structural analyses, such as those based on crystal and cryomicrographic data (PDB IDs: 3V0B and 3WIN), the BoNT/A and BoNT/B complexes exhibit a triskelion-like structure (Figure 2B), where BoNT/A directly binds to NTNHA. NTNHA is further associated with three flexible appendages formed by the HA proteins (HA70, HA17, HA33). Notably, analysis by [9] identified additional HA proteins in the BoNT/A complex—HA48 and HA23—which are the result of HA70 cleavage.

Among the various neurotoxin complex proteins, NTNHAs are the most highly conserved, displaying a high degree of sequence identity (~66–83%). For instance, NTNHA of BoNT/A shares 82.9%, 65.8%, 65.9%, 65.8%, 74.9%, and 72.2% sequence identity with NTNHA from BoNT serotypes B, C, D, E, F, and G, respectively [9]. Based on these high sequence identities, it can be inferred that NTNHAs across different BoNT serotypes share similar structural organizations. However, despite the structural similarities, BoNTs themselves show a much lower amino acid sequence identity (~20%) among the different serotypes.

Both botulinum neurotoxin A (BoNT) and neuronal-toxin-associated proteins (NTNHAs) share structural similarities, particularly in their motifs and zinc-binding sites. The catalytic domain of BoNT contains the well-characterized HEXXH + E motif, where the first histidine (His) and the second glutamate (Glu) coordinate a Zn^2^^+^ ion, while the first glutamate coordinates a water molecule essential for hydrolysis, a critical step in BoNT’s enzymatic activity [12,13]. In contrast, NTNHA proteins feature a non-catalytic KCLIK motif that is structurally similar but does not bind zinc, highlighting the functional divergence between the two [14]. These observations suggest that BoNT and NTNHA share a common evolutionary ancestor, with both proteins possibly originating from a similar structural framework.

Another noteworthy feature of BoNT/A and its associated neurotoxin-associated proteins (NAPs), including NTNHA, is their increased proteolytic resistance. When BoNT is complexed with NTNHA and other NAPs, it exhibits enhanced stability against protease degradation compared with the toxin alone [12,13,14,15]. This protective association is particularly important for the toxin’s stability during its transit through the gastrointestinal tract and may contribute to its ability to evade the host immune response, thus increasing the efficiency of toxin delivery.

NTNHA proteins, such as NTNHA/A, NTNHA/C, and NTNHA/D, also feature a self-nicking site between Lys133 and Lys134. This site leads to the formation of two polypeptide chains (~125 kDa and 15 kDa), which remain non-covalently bound together [13]. A critical structural distinction between NTNHA and BoNT/A is the absence of the long flexible loop connecting the light chain (LC) and heavy chain (HC) in BoNT/A. This loop contains a post-translational nicking site, cleaved by an endogenous enzyme to activate the toxin. The flexible loop in BoNT/A is believed to play a role in the translocation and release of the light chain into the cytosol, a process essential for the toxin’s toxicity. In contrast, NTNHA does not contain a homologous loop or any disulfide bond, suggesting that NTNHA does not undergo similar activation mechanisms to BoNT. Additionally, NTNHA maintains a belt region (Asn493–Asp546 in BoNT/A; Asp451–Asn496 in BoNT/B), a structural feature also presents in BoNT/A, where it acts as a pseudo-inhibitor [13]. However, the precise function of the belt in NTNHA is still unclear, as it does not seem to play the same inhibitory role seen in BoNT/A, where it prevents premature enzymatic activity.

The structural similarities and differences between NTNHA and botulinum neurotoxin (BoNT) raise several interesting questions, particularly regarding their functional interactions and evolutionary origins. The high sequence identity between NTNHA and BoNT, along with their similar structural architectures, suggests that NTNHA may share some characteristics with BoNT, but it also presents unique features. The following questions are worth addressing:

- Binding specificity across serotypes: Given the high sequence identity and probable structural similarity between NTNHA proteins, could NTNHA from one BoNT serotype interact similarly with botulinum neurotoxin from another serotype, or is this interaction highly specific? This is a critical question because the interaction between NTNHA and BoNT may be influenced by subtle structural differences, which could affect the cross-serotype functionality.
- High sequence similarity of NTNHA: Why does NTNHA exhibit such a high sequence similarity to BoNT? This raises questions about the evolutionary pressures that led to such a conserved structure. Is this similarity a result of a shared function, or is it indicative of an ancestral origin?
- Biological function of NTNHA: Since NTNHA lacks catalytic activity, what is its biological function? Despite its structural resemblance to BoNT, NTNHA does not participate in the enzymatic cleavage of substrates. Its role likely lies in supporting the stability, transport, or protease resistance of the neurotoxin complex, but further studies are needed to fully elucidate its function.
- Evolutionary divergence in catalytic activity: Why did one component of the BoNT complex (BoNT) acquire metalloprotease activity, while a similar structural component (NTNHA) lacks this activity? This raises intriguing questions about the evolutionary forces that led to functional divergence despite similar structural motifs.
- BoNT-NTNHA complex as a toxin–antitoxin system: Although the BoNT-NTNHA complex resembles a toxin–antitoxin system, its biological behavior does not align with traditional toxin–antitoxin systems. Why is this the case? While toxin–antitoxin systems typically involve an equilibrium between a toxic protein and its inhibitor, the interaction between BoNT and NTNHA appears to serve different functional roles, particularly in protecting the toxin from protease degradation and aiding in its transport.
- Absence of a Zn-binding motif in NTNHA: Why does NTNHA, despite its structural similarity to BoNT, lack the Zn^2^^+^-binding motif? The absence of the HEXXH motif in NTNHA raises questions about its functional divergence from BoNT. This structural variation may be a key aspect of NTNHA’s non-catalytic role and its interaction with BoNT. The purpose of this motif’s absence in NTNHA may relate to its role as a stabilizing or protective partner rather than an active enzymatic component.
- NTNHA as a non-neuronal binding protein: Why is NTNHA not a neuronal binding protein? Despite its structural similarity to the heavy chain of BoNT, which is involved in binding to neuronal cells, NTNHA does not exhibit similar binding characteristics. This suggests that NTNHA’s role in the BoNT complex may be distinct from the toxin’s interaction with neuronal membranes, potentially focusing on stability or other functions that do not involve direct neuronal binding.

## 4. Evolutionary Insights: BoNT/NTNHA Gene Cluster

The BoNT/NTNHA gene cluster is not widely distributed in nature and is primarily found in Clostridia. However, recent studies have identified a similar gene cluster in the Bacillus sp. isolate 2SH. This cluster encodes a protein, BNA (BoNT/NTNHA-like component A), which shares significant structural features with both BoNT and NTNHA. The predicted 825-amino acid sequence of BNA contains the catalytic and translocation domains typical of Clostridial neurotoxins but lacks the C-terminal domain of the BoNT heavy chain and NTNHA [16]. BNA also shares several conserved motifs with BoNT and NTNHA, such as:

- RXXY (active-site stabilizing motif);
- PWISQSLN (translocation motif);
- Two cysteine residues, C376 and C386, located between the translocation and catalytic domains (similar to the disulfide bond found in BoNT).

Interestingly, BNA lacks the HEXXH motif found in BoNT but contains the sequence SKLIE, whose functional significance remains unclear. The predicted 3D structure of BNA suggests that it has structural similarities to the light chain and translocation domains of both BoNT and NTNHA, which implies that it may be capable of translocating into host cells. However, it is likely to lack protease activity and may not have specificity for neuronal cells [16]. This finding is significant because it demonstrates that BoNT-like or NTNHA-like domains may have been exchanged between different complexes during evolutionary processes. The presence of such domains in other organisms, like Bacillus sp., provides preliminary evidence that BoNT or NTNHA may have been incorporated into the neurotoxin complex during evolution, suggesting a more complex evolutionary history than previously understood.

## 5. HA Proteins and Their Role in BoNT Complexes

Hemagglutinin (HA) proteins are the most abundant proteins in the HA(+) BoNT complexes. These proteins exhibit hemagglutination activity by binding to specific oligosaccharides present on the surface of host cells. For instance, HA33 of BoNT/A binds to glycolipids and glycoproteins on erythrocyte membranes that contain the Galβ1-4GlcNAcmotif (galactose-β1-4glucose-N-acetyl-D-neuraminic acid) [17]. On the other hand, HA33 from BoNT types C and D requires Galβ1-4GlcNAc as well as sialylglycolipids (e.g., GM3) and sialoglycoproteins with the N-acetyl-D-neuraminic acid-α2-3-galactose-β1 motif for binding [18,19].

HA33 of the BoNT/A complex is a dimeric β-sheet protein [20] and undergoes post-translational modifications. Notably, the N-terminal region of HA33 is cleaved, with the first five amino acids removed, a modification not observed in the HA33 protein from Clostridium botulinum type C. Additionally, the C-terminal region of HA33, consisting of 31 amino acids, is crucial for its hemagglutination activity due to the carbohydrate recognition site. Conserved residues such as Asp263 and Asn285 are critical for the binding of HA33 to carbohydrates [20].

HA17, another component of the BoNT complex, adopts a β-trefoil structure (similar to that of the ricin B-chain or the C-terminus of the BoNT heavy chain). However, the precise function of HA17 remains unclear. HA48 and HA70 are also involved in carbohydrate recognition, specifically binding to sialosylparagloboside and GM3 on erythrocyte membranes through the N-acetyl-D-neuraminic acid-α2-3-galactose-β1 motif [19]. HAs play an important role in the stability of the BoNT complex, which is critical for oral infection [21]. Additionally, they have been shown to disrupt epithelial barriers, contributing to pathogenesis [22].

## 6. Interaction with E-Cadherin

HA proteins recognize E-cadherin, a key adhesion molecule found on the surface of epithelial cells. However, their interaction with E-cadherin is species- and isoform-specific. For example, BoNT-associated HAs specifically recognize epithelial E-cadherin but do not interact with neuronal or vascular isoforms of E-cadherin. Importantly, individual HA proteins are not capable of binding to E-cadherin on their own; the entire HA complex, comprising HA17, HA33, and HA48/70, is required for optimal binding with high affinity.

## 7. OrfX Proteins and Their Role in BoNT Complex Formation

Despite the lack of structural similarity between OrfX and HA proteins, their proximity within the BoNT gene cluster suggests a potential role in the formation of the toxin complex and its pathogenesis [23]. However, the precise function of OrfX remains to be elucidated. Unlike HAs, OrfX does not interact with E-cadherin; instead, OrfX1 and OrfX2 bind to phosphatidylinositol, indicating a distinct role in cellular interactions [23].

Interestingly, BoNT serotypes or subtypes that are Orf(+)HA(−) are also responsible for foodborne botulism. Initially, it was believed that the OrfX-P47 gene clusters were exclusive to BoNT-producing organisms. However, it has since been shown that OrfX-P47 gene clusters are widely distributed across bacterial and insect genomes [16]. This suggests that the OrfX-P47 genes may not be specifically associated with BoNT pathogenesis. Instead, their primary role might involve bacterial and insect pathogenesis, potentially influencing processes such as the bacterial cell wall envelope, oral toxinogenesis, or the release and trafficking of toxins.

## 8. Regulatory Role of OrfX

Recent studies by [24] suggest that OrfX1 or P47 may act as regulatory elements in the BoNT complex. Structurally, OrfX contains a tubular lipid-binding (TULIP) domain, which is associated with lipid binding, further suggesting that OrfX might participate in membrane interactions or lipid-related processes. OrfX has also been shown to interact with NTNH/E, with the binding interface overlapping with the HA-70 binding region on NTNH/A [24]. This interaction could play a role in the stability or trafficking of the toxin complex.

## 9. Interaction of Botulinum Toxin with the Epithelial Barrier

Botulism is primarily a foodborne disease, and the toxin typically does not penetrate intact skin. Naturally occurring foodborne botulism is associated with nausea, abdominal cramps, vomiting, and diarrhea. Upon ingestion, the toxin is protected by neurotoxin-associated proteins (NAPs), which shield it from the harsh acidic environment and proteolytic degradation in the digestive tract. When taken orally, the BoNT complex (including its NAPs) is 10 to 1000 times more toxic than the isolated neurotoxin. For the toxin to exert its biological effects, it must navigate a long and difficult journey through the digestive system.

1. **First Hurdle:** The Harsh Environment of the Digestive Tract

The first obstacle is the harsh conditions of the digestive tract, specifically the acidic pH. However, the toxin complex is protected by NTNHA and HAs. While the toxin complex is typically stable at pH 6.5 and thought to dissociate above pH 7.0, Sakaguchi et al. (1977) [25] reported that the BoNT complex remains stable in the digestive tract, aiding its protection during its passage through the stomach.

2. **Second Hurdle:** The Epithelial Barrier

The second challenge is the intestinal epithelial barrier, which the toxin must cross to reach the enteric nervous system (ENS). Sugii et al. [25,26] demonstrated the absorption of the entire BoNT complex from the intestinal lumen into the lymphatic system in rats. However, the complex is believed to dissociate in a near-neutral pH buffer. Several studies suggest that gangliosides (GD1b, GT1b) and the SV2C receptor on intestinal epithelial cells may act as potential binding sites for BoNT [27]. BoNT/A enters via a Cdc42-dependent, clathrin-independent pathway, distinct from the case in neuronal cells, where BoNT typically enters through a clathrin-dependent pathway. After entering the early endosomal compartment, BoNT/A undergoes further processing. The 16S form of BoNT (rather than the 12S form) selectively binds and enters intestinal cells via sialic acid-containing O-linked glycoproteins on the cell surface [28]. Once inside the epithelial cells, the toxin is transported to the Golgi apparatus. Interestingly, the non-toxic components (e.g., HAs) are not required for BoNT transport in cultured epithelial cells [29,30]. However, it is well established that HAs in the BoNT complex facilitate transcytosis and vesicular trafficking across the intestinal epithelium [18,19].

3. **Disruption of the Epithelial Barrier by HAs**

HAs, particularly HA17, HA33, and HA48/70, bind to intestinal epithelial cells and disrupt the tight junctions between these cells. Specifically, HAs loosen the binding of tight-junction proteins such as occludin, ZO-1, β-catenin, and E-cadherin without significantly affecting the viability of the cells [22]. Interestingly, all three HAs are required for efficient binding and the disruption of the epithelial barrier, as this is consistent with the behavior of other pathogens like Listeria monocytogenes, Candida albicans, Bacteroides fragilis, and Porphyromonas gingivalis, all of which utilize E-cadherin-mediated adhesion for host cell internalization [31,32].

Upon dissociation from the toxin complex in the endosomal compartment, HA proteins are transcytosed to the basolateral surface of epithelial cells, where they interact with E-cadherin. This binding interferes with E-cadherin dimerization, which is critical for maintaining tight-junction integrity. As a result, the toxin is able to cross the intestinal barrier through paracellular transport, facilitated by the disruption of tight junctions by HAs.

4. **Efficient Transport Across Epithelial Cells**

The passage of BoNT through intestinal epithelial cells is not highly efficient, with only 1% or less of the toxin crossing the epithelium [27,28,29]. This rate is consistent with other bacterial or viral enteric pathogens, which exhibit transport rates in the range of 0.1–10% [33,34]. The transport is temperature-dependent, occurring efficiently at 37 °C but blocked at 4 °C. Interestingly, BoNT is transported less efficiently in epithelial cells compared with neuronal cells. In neuronal cells, BoNT-containing vesicles preferentially traffic from the cell surface to the perinuclear region, whereas in intestinal cells, these vesicles are more scattered in the cytosol. This differential trafficking may be due, in part, to the lower abundance of SV2 and related proteins in intestinal cells compared with neuronal cells, which could influence BoNT binding and internalization [27,28,29,35,36]. The possibility of other receptors contributing to BoNT binding in epithelial cells, or a steric effect preventing efficient uptake by SV2, warrants further investigation.

5. **Retrograde Transport and Interaction with the Enteric Nervous System**

Once BoNT crosses the intestinal barrier, it diffuses and interacts with the enteric nervous system (ENS), which is distributed across two regions: the submucosal and myenteric plexuses. The ENS controls various gastrointestinal functions, and about 55% of neurons in the submucosal plexus and 80% of neurons in the myenteric plexus are cholinergic, making them the primary targets of BoNTs. The submucosal plexus regulates ion secretion and sensory pathways, while the myenteric plexus controls intestinal motility. In addition to cholinergic neurons, the ENS contains other neuronal types, such as VIP (vasoactive intestinal peptide) neurons and serotonergic neurons, which regulate smooth muscle relaxation and fluid secretion. The effect of BoNT on these non-cholinergic neurons is still not fully understood [37].

Interestingly, the ENS and central nervous system (CNS) are highly interconnected, suggesting that BoNT could use retrograde transport to affect CNS neurons. The retrograde transport mechanism of BoNT has been partially established in several studies [38,39,40,41], and it is likely that this pathway contributes to the systemic effects of botulism.

## 10. Interaction of Botulinum Toxin with Neuronal Cells

After entering the lymphatic and blood circulation, either through intestinal absorption, inhalation, or injection, botulinum toxins (BoNTs) quickly reach the perineuronal fluid, where they can exert their toxic effects. However, BoNTs do not cross the blood–brain barrier (BBB) [42]. Their primary site of action is the nerve terminal, where they bind with high affinity and specificity, which contributes to the potent neurotoxic effect of the toxin. BoNTs utilize a dual-receptor mechanism to bind to neuronal cells, which involves gangliosides and synaptic vesicle protein 2 (SV2). Gangliosides are glycosphingolipids present on the surface of neuronal membranes, and they play a critical role in BoNT binding. The binding of BoNT to gangliosides occurs through a conserved motif, E..H..SXWY..G, with some variations depending on the BoNT serotype. For instance, BoNT/E has the motif E..K..SXWY..G, while for BoNT/G, it is G..G..SXWY..G [43,44]. Studies on ganglioside knockout mice have shown decreased sensitivity to clostridial neurotoxins, confirming the importance of gangliosides in BoNT binding [45,46,47,48]. Gangliosides such as GD1b, GT1b, and GQ1b are the key receptors for BoNT binding (Table 2). Gangliosides are abundant on neuronal cell membranes, especially in motor neurons, but they are also present in other tissues, though at much lower concentrations in the intestinal mucosa. This difference in ganglioside abundance likely explains the higher neuronal selectivity of BoNTs. In addition to gangliosides, lipids like phosphatidylethanolamine and carbohydrates like sialic acid contribute to the functional receptor complex for BoNTs [49]. Gangliosides are integral to various neuronal functions, including myelin formation, axon stability, and the organization of ion channels at the nodes of Ranvier [50,51,52].

After entering into the lymphatic and blood circulation, either through intestinal adsorption or inspiration or injection, the BoNTs quickly access the perineuronal fluid. However, BoNTs do not cross the blood–brain barrier (BBB; Simpson, 2013). BoNTs bind with very high affinity and specificity to the nerve terminal, which is the basis of its high potency.As mentioned earlier, BoNT utilizes a dual-receptor mechanism for binding to neuronal cells that involves gangliosides and SV2 (synaptic vesicle protein 2). In addition to membrane proteins, lipids like phosphatidylethanolamine, along with a carbohydrate (sialic acid; together they form part of the ganglioside receptor), were demonstrated to be the functional receptors of BoNTs (Tsukamoto et al., 2005). Gangliosides are present in all types of vertebrate tissues (there are no gangliosides in invertebrates except in sea urchins); however, they are abundant in the neuronal cell membrane. They are involved in the binding of myelin-associated glycoprotein (MAG) to neurons, myelin formation, axon–myelin interactions, peripheral and central axon stability, organization of ion channels at nodes of Ranvier [50,51,52]. Notably, GD1a is more abundant in motor neurons compared with sensory neurons. While gangliosides are present throughout various tissues, their concentration is significantly higher in neuronal cells, particularly in motor neurons, which is essential for BoNT’s high neuronal selectivity. Gangliosides are plasma membrane receptors. Among the gangliosides found in the human brain and peripheral nervous system (PNS), four main types have been identified: GM1, GD1a, GD1b, and GT1b. The intestinal mucosa has a lower prevalence of gangliosides (400–700 fold less) than in neurons, and this is one of the reasons for the higher neuronal selectivity of BoNTs.

While gangliosides are critical for BoNT binding, they are not the only receptors for the toxin, as BoNTs do not bind to non-neuronal cells containing gangliosides. In addition to gangliosides, synaptic vesicle proteins (SV2) have been identified as key receptors for several BoNT serotypes.

SV2 was first identified as a receptor for BoNT/B by [52,53]. Later, SV2 was also shown to be a receptor for BoNT/DC and BoNT/G [54,55,56,57,58].

BoNT/A, BoNT/E, BoNT/D, and BoNT/F also utilize SV2 as a receptor [54,55,56,57,58]. Interestingly, glycosylation of the SV2 receptor enhances BoNT binding, further emphasizing the importance of SV2 in toxin interaction.

The ganglioside-binding site is located at the C-terminal side of the heavy chain (HC_C_; 25 kDa of c-terminus of HC domain), in addition the SV2-binding site is also located in the HC_C_ domain. The function of the HC_N_ domain is still unknown [44]; however, it does form an interface with the HC_C_ domain. BoNT/C utilizes dual ganglioside receptors to facilitate its cell entry [58] (Table 2).

SV2 (synaptic vesicle protein 2) is a transmembrane protein expressed in both central and peripheral neurons as well as in endocrine cells but not in exocrine cells. There are three isoforms of SV2: SV2A, SV2B, and SV2C. SV2A and SV2B are widely distributed in the brain, while SV2C has a more restricted distribution. Despite this, SV2C is expressed in all types of neurons, including GABAergic, cholinergic, and dopaminergic neurons. In contrast, SV2A is predominantly associated with glutamatergic and GABAergic neurons, while SV2B is primarily linked to glutamatergic neurons. In the peripheral nervous system, all three isoforms are present. BoNTs interact with SV2 in two forms: the free peptide form and the N-glycosylated protein form. The ability of BoNTs to bind both forms of SV2 is a key factor in their exceptional neuronal specificity and high potency. Interestingly, it is the binding to SV2, rather than gangliosides, that determines how many molecules of BoNT enter the neuronal cells [59]. Colasante et al. [60] demonstrated that for BoNT/A, internalization and trafficking inside the neuronal cytosol primarily involve synaptic vesicles, not the endosomal compartment. This suggests that the light chain (LC) of the toxin enters the cytosol very rapidly after endocytosis, although the possibility of retrograde transport has not been ruled out.

Another critical BoNT receptor is synaptotagmin (Syt), a calcium-dependent protein involved in synaptic vesicle fusion. Syt has four domains: an N-terminal transmembrane domain, a small lumen domain, and two long cytoplasmic domains. Syt acts as a calcium sensor that triggers the fusion of synaptic vesicles with the plasma membrane. There are 17 known isoforms of Syt, but Syt I and Syt II have been shown to be involved in exocytosis, with Syt II being more abundant than Syt I in motor neurons. Syt is also widely distributed in the brain, including in excitatory and inhibitory neurons. The affinity of BoNT/B for the nerve terminal is highly dependent on both GT1b (ganglioside) and Syt concentrations [52,53,61,62,63,64].

Botulinum toxin (BoNT) primarily internalizes into cells via the endocytic pathway, although alternative hypotheses suggest that retrograde pathways and other mechanisms may also play a role in BoNT internalization. Beyond its well-known inhibition of acetylcholine release, BoNT can also interfere with the release of several other neurotransmitters, hormones, and neuropeptides [41,65]. However, certain neuropeptides—such as neuropeptide Y, vasointestinal peptide (VIP), and calcitonin-gene-related peptide (CGRP)—are not inhibited by BoNT release from afferent neurons or sweat glands [50,66,67]. Motor neurons are more sensitive to BoNT compared with GABAergic, dopaminergic, and glutamatergic neurons. This higher sensitivity is attributed to the greater abundance of BoNT receptors and the high synaptic activity in motor neurons.

After internalization and translocation to the cytosol, BoNT targets SNARE proteins, which are essential for the fusion of synaptic vesicles with the plasma membrane during neuroexocytosis. SNARE proteins are a large family, consisting of isoforms like SNAP-25, VAMP, and syntaxin (Table 3). These proteins form a heterotrimeric complex that is resistant to BoNT and that is crucial for neurotransmitter release. Interestingly, SNAP-25 exists in multiple pools within the nerve terminal, but only 10–15% of these pools are involved in neuroexocytosis [68]. BoNT selectively targets these 10–15% of SNARE proteins, resulting in the paralysis of the affected neuron [69].

While BoNT’s primary targets are neuronal cells, its receptors and target proteins are also found in non-neuronal cells. The sensitivity of BoNT in these cells varies based on the concentration of receptors and the specific internalization pathways that the toxin must use to exert its effects. In non-neuronal cells, signaling release can occur via two mechanisms: SNARE-dependent and SNARE-independent pathways. BoNT primarily inhibits the SNARE-dependent mechanism, but microarray data suggests that it may also influence other cellular signaling pathways, including phosphatidylinositol signaling, complement and coagulation pathways, and calcium signaling [70]. Additionally, BoNT inhibits the release of catecholamines from chromaffin cells, ATP and glutamate from glial cells, insulin from β-pancreatic cells, and TNF from monocytes. It also cleaves VAMP2 in astrocytes and NAD in human bladder cells [52,53].

A variety of non-neuronal cells are known to express BoNT receptors or the intracellular targets of BoNT, including epidermal keratinocytes, mesenchymal stem cells, intestinal cells, prostate epithelial cells, alveolar epithelial cells, breast cell lines, dermal fibroblasts, mast cells, sebocytes, vascular endothelial cells, neutrophils, and macrophages [71]. The expression levels of BoNT receptors vary across these cell types. Furthermore, BoNT/A can cleave SNAP-23, a SNARE protein that is ubiquitously expressed in human tissues, adding to the toxin’s broad cellular impact.

The interaction mechanism of BoNT with non-neuronal cells differs from that with neuronal cells. In neuronal cells, BoNT typically internalizes via a clathrin-dependent pathway, whereas in intestinal cells, BoNT uses a Cdc42-dependent pathway in combination with the binding of HA proteins (HA17, HA33, and HA70) to E-cadherin to facilitate internalization (as discussed previously).

In naturally occurring botulism, symptoms related to autonomic nervous system dysfunction are commonly observed. These include disturbances in secretion, such as dry mouth, dry throat, xerophthalmia (dry eyes), urinary retention, and intestinal hypomotility [72]. These symptoms may arise from the direct or indirect effects of botulinum toxin (BoNT) on the autonomic nervous system. Specifically, BoNT/A has been shown to block secretion at the cholinergic terminals of both pre- and post-ganglionic nerve terminals within the parasympathetic and sympathetic nervous systems [73]. Despite its effects on the cholinergic autonomic system, the heart is only moderately affected by BoNT. This is because the cholinergic neurons of the heart are primarily parasympathetic, whereas the catecholaminergic neurons involved in the heart’s sympathetic regulation are less susceptible to BoNT’s action.

**Table 3:** Cleavage sites of BoNTs (modified from Pirazzini et al. [74]. Some of these sites were determined experimentally, while others are predicted through sequence alignment. For further details, please refer to Pirazzini et al. [74] and Kumar et al. [3] for more information on cleavage site alignments. ND: Not Determined.

Serotype	Species	Cleavage Site of Substrate		
		Mouse	Rat	Human
BoNT/A	SNAP23	T202–R203	T202–R203	A203–R204
	SNAP25a/b	Q197–R198	Q197–R198	Q197–R198
	SNAP29	K255–K256	K253–K254	R253–K254
	SNAP47	R407–R408	R413–R414	R459–R460
BoNT/B	VAMP1	Q78–F79	V78–F80	Q78–F79
	VAMP2	Q76–F77	Q76–F77	Q76–F77
	VAMP3	Q63–F64	Q63–F64	Q63–F64
	VAMP4	A98–F99	A98–F99	A98–F99
	VAMP5	A51–F52	A51–F52	T51–F52
	YKT6	A183–F184	A183–F184	A183–F184
	VAMP7	T171–F172	T171–F172	T171–F172
	VAMP8	H58–F59	H59–F60	H58–F59
	SEC22	K180–Y181	K180–Y181	K180–Y181
BoNT/C and BoNT/CD	SNAP23	R203–A204	R203–A204	R203–A204
	SNAP25 a/b	R198–A199	R198–A199	R198–A199
	SNAP29	K256–V257	K254–V255	K254–V255
	SNAP47	R408–M409	A414–M415	A459–M460
	STX1A	K253–A254	K253–A254	K253–A254
	STX1B	K252–A253	K252–A253	K252–A253
	STX2	K253–A254	K254–A255	K252–A253
	STX3	R253–A254	R253–A254	K253–A254
	STX4	I261–A262	I261–A262	I261–A262
	STX5	K324–Y325	K324–Y325	K324–Y325
	STX6	K224–V225	K224–V225	K224–V225
	STX7	R226–M227	R226–M227	R226–M227
	STX8	L206–V207	L206–V207	L206–V207
	STX11	K265–A266	K265–A266	K265–A266
	STX12	R239–A240	R239–A240	R239–A240
	STX16	K292–A293	K292–A293	K292–A293
	STX17	K222–A223	K222–A223	K222–A223
	STX18	E203–A204	E203–A204	E203–A204
	STX19	L268–A269	ND	R270–L271
BoNT/E	SNAP23	K185–I186	K185–I186	R185–I186
	SNAP25 a/b	R180–I81	R180–I81	R180–I81
	SNAP29	R238–L239	R236–L237	R236–L237
	SNAP47	G390–I391	G396–I397	G441–V442
BoNT/F5 and BoNT/FA	VAMP1	L56–E57	L56–E57	L56–E57
	VAMP2	L54–E55	L54–E55	L54–E55
	VAMP3	L41–E42	L41–E42	L41–E42
	VAMP4	I76–E77	I76–E77	I76–E77
	VAMP5	L29–E30	L29–E30	L29–E30
	YKT6	L1622–E163	L162–E163	L162–E163
	VAMP7	A149–Q150	A149–Q150	A149–Q150
	VAMP8	L36–S37	L36–S37	L36–S37
	SEC22	L58–Q59	L58–Q59	L58–Q59
BoNT/F1	VAMP1	Q60–K61	Q60–K61	Q60–K61
	VAMP2	Q58–K59	Q58–K59	Q58–K59
	VAMP3	Q45–K46	Q45–K46	Q45–K46
	VAMP4	E80–R81	E80–R81	E80–R81
	VAMP5	G60–K61	G60–K61	V60–K61
	YKT6	E166–K167	E166–K167	E166–K167
	VAMP7	E153–R154	E153–R154	E153–R154
	VAMP8	E40–N41	E39–N40	E40–N41
	Sec22	E162–A163	E162–A163	E162–A163
BoNT/G	VAMP1	A83–A84	A83–A84	A83–A84
	VAMP2	A81–A82	A81–A82	A81–A82
	VAMP3	A68–A69	A68–A69	A68–A69
	VAMP4	S103–K104	S103–K104	S103–K104
	VAMP5	T56–K57	T56–K57	T56–Q57
	YKT6	A189–R190	A189–R190	A189–R190
	VAMP7	S176–R177	S176–R177	S176–R177
	VAMP8	S63–Q64	S63–Q64	S63–Q64
	Sec22	A185–K186	A185–K186	A185–K186
BoNT/D and BoNT/DC	VAMP1	K61–L62	K61–L62	K61–L62
	VAMP2	K59–L60	K59–L60	K59–L60
	VAMP3	K46–L47	K46–L47	K46–L47
	VAMP4	R81–L82	R81–L82	R81–L82
	VAMP5	K61–L62	K61–L62	K61–L62
	YKT6	K167–L168	K167–L168	K167–L168
	VAMP7	R154–L155	R154–L155	R154–L155
	VAMP8	N41–L42	N41–L42	N41–L42
	Sec22	A163–L164	A163–L164	A163–L164

## 11. Trafficking in the CNS

Botulinum toxin (BoNT) is well known for its action on peripheral neurons, where it blocks the release of acetylcholine, leading to muscle paralysis. However, not all clinical effects of BoNT can be attributed to this mechanism alone. Although there is a lack of direct experimental evidence from in vivo models demonstrating the CNS effects of BoNT, clinical observations provide some support for the hypothesis that BoNT may act beyond the neuromuscular junction. Recent evidence challenges the previous assumption that BoNT remains confined to the axon terminal’s active zone near the plasma membrane. Instead, clinical data suggest that BoNT’s effects extend to the spinal and supraspinal levels. For example, BoNT injections have been shown to improve muscle tone in untreated areas of the body, suggesting a more widespread impact [75,76].

BoNT is generally more active in excitatory neurons than in inhibitory neurons, potentially due to the lower levels of intracellular targets in inhibitory neurons [77]. Interestingly, BoNT/A2 appears to be more effective in inhibitory neurons than in excitatory neurons [78]. This differential effect, coupled with the clinical observations, suggests that BoNT may have an impact on CNS neurons.

Restani et al. [79] demonstrated that BoNT/A could impair neurotransmission from the retina to the tectum via retrograde axonal transport and transcytosis. Beyond its effects on neuronal function, BoNT has been shown to contribute to the reorganization of the central circuitry [80]. Structural abnormalities in the thalamus, sensorimotor cortex, and basal ganglia were observed after BoNT treatment, although these changes were reversible within four weeks [81]. Further studies have reported changes in gray matter volume and cortical morphology following BoNT treatment [82,83]. These structural changes are thought to be both BoNT-dependent and disease-dependent, indicating that the long-term effects of BoNT on the CNS remain to be fully understood. However, it is increasingly clear that the peripheral action of BoNT at the neuromuscular junction does not fully explain its central effects [84,85].

An intriguing finding is that the unilateral administration of BoNT can produce bilateral anti-nociceptive effects. This observation supports the hypothesis of retrograde transfer, wherein BoNT is transferred from motor neurons to the spinal cord via transcytosis to secondary spinal neurons. In a rat model, BoNT was shown to undergo long-distance transport after peripheral injection, with cleaved SNAP-25 detected in spinal cord motor neurons. Inhibition of retrograde transport by colchicine blocked the nociceptive effects, further confirming the involvement of retrograde transport in BoNT’s action [86,87,88].

Given the specificity of BoNT for neuronal circuitry, several scenarios may explain its effects on the CNS:

- Peripheral BoNT injections produce indirect effects on the CNS.
- BoNT may utilize alternative internalization pathways, such as a retrograde pathway followed by transcytosis to reach secondary neurons.
- Receptors and targets for BoNT are present in CNS neurons, meaning that the direct administration of BoNT could have therapeutic effects.
- Non-neuronal cells may also be affected by BoNT, indirectly altering the neuronal circuitry.
- Peripheral SNARE cleavage could trigger pathways leading to the degradation, reduced expression, or inhibition of neurotransmitter release in distal neurons.

A significant study by [89] demonstrated the indirect effects of BoNT/A on sensorimotor activation beyond the circuits controlling the treated body part in cervical dystonia. Using fMRI, the study showed that BoNT’s effects extended to bilateral areas of the brain, including the primary and secondary somatosensory cortices, parietal lobules, premotor cortex, cingulate cortex, and thalamus. Similar observations have been made in other studies [90,91,92,93], where BoNT administration altered brain connectivity, increasing activity in some regions while decreasing it in others. These studies also noted changes in sensory processing in cervical dystonia patients following BoNT injection.

Additionally, BoNT administration has been associated with improvements in memory and learning, suggesting a role for BoNT in neuronal plasticity [94]. Yesudhas et al. [94] found that intramuscular injection of BoNT enhanced hippocampal plasticity and improved cognitive functions, although the exact mechanisms are still unclear. These findings underscore the potential indirect central effects of BoNT following peripheral administration. The hypothesis of retrograde transfer of BoNT is further supported by studies detecting cleaved SNAP-25 in CNS neurons following peripheral injection. Papagiannopoulou et al. [95] showed that radiolabeled BoNT/A was distributed to CNS neurons (lumbosacral dorsal root ganglion) after bladder injections in rats. The detection of cleaved SNAP-25 in spinal astrocytes suggests that BoNT may also affect non-neuronal glial cells, potentially via transcytosis to the spinal cord [96]. Immunostaining also revealed cleaved SNAP-25 colocalized with GFAP (a glial marker), CD11b (a microglial marker), and NeuN (a neuronal marker), supporting the idea of BoNT action on glial cells in addition to neurons. Koizumi et al. [97] showed that BoNT/A, injected into the gastrocnemius muscle, could spread to the contralateral spinal cord, with cleaved SNAP-25 detected. They found that BoNT/A1 was more effective in spreading than the A2 subtype. The exact mechanism of retrograde transport remains unclear, but it is likely that BoNT utilizes microtubule motors or axon signaling endosomes to reach the soma, from where it can be released into the extracellular space to act on secondary neurons [39].

Direct administration of BoNT into the CNS has been associated with neuroprotective effects, including antidepressant effects, enhancement of brain-derived neurotrophic factor (BDNF), increased antioxidative enzyme activity, and decreased activity of glutathione and glutathione peroxidase [98,99,100]. Most CNS studies have utilized BoNT/A, but BoNT/B has also shown promise in reducing substance P release, spinal c-Fos expression, and pain in mice models [101].

BoNT’s potential for treating neurological disorders is evident, as seen in Alzheimer’s disease (characterized by cholinergic neuron loss) and Parkinson’s disease (characterized by dopaminergic neuron loss). In animal models, BoNT/A administration improved motor dysfunction without affecting cognitive or cholinergic functions [102,103,104]. On the other hand, BoNT/B has been used to model dementia with cognitive defects [105]. These findings highlight the therapeutic potential of BoNT, either as a direct therapeutic agent or as a vehicle for targeted delivery to the CNS.

## 12. Conclusions

The mechanism of botulinum toxin (BoNT) infection remains an area of active research, and while we have gained significant insights into its actions, many questions remain unresolved. Initially, its mechanism of action was understood through comparisons with other toxins and related molecules. However, BoNT’s unique structural features, its internal flexibility, and the intricate relationship between its structure and function make it an exceptionally interesting molecule for both basic and applied research, as well as for therapeutic applications.

BoNT’s high potency, specificity, and longevity can be attributed to its complex and multifaceted mechanism of action. This complexity gives rise to several intriguing questions and challenges that warrant further investigation. Some of these questions include:

- *Inhibition of acetylcholine release before SNAP-25 cleavage:* It has been shown that acetylcholine release is inhibited before the detection of SNAP-25 cleavage. This raises the question: How is acetylcholine release inhibited before SNAP-25 cleavage occurs?
- *Central effects after peripheral administration:* Evidence suggests that BoNT has central effects following peripheral administration, but the mechanisms underlying this action remain unclear. Understanding how BoNT affects the central nervous system (CNS) after being injected into peripheral sites is crucial.
- *Longevity of BoNT inside the cell:* The duration of BoNT inside the cell, particularly how long it remains active after internalization and translocation to the cytosol, is still under investigation. What mechanisms allow BoNT to exert prolonged effects once inside the cell?
- *Other effects beyond acetylcholine inhibition:* While BoNT is primarily known for inhibiting acetylcholine release, there may be other effects of BoNT administration that have not been fully explored. Does BoNT affect other signaling pathways or cellular functions beyond its well-known impact on neurotransmitter release?
- *Transport of BoNT through the gut:* Since botulism is primarily a food-borne disease, the transport of BoNT through the gut needs to be studied in more detail. Additionally, the potential effects of BoNT on the gut–brain axis could provide new insights into its systemic impacts and open up new avenues of research.

Investigating these challenges will not only answer critical questions about the toxin’s actions but also uncover new scientific perspectives related to its cellular mechanisms and systemic events. Such research could lead to important advances in understanding how BoNT functions at multiple levels, from the molecular to the systemic, and could ultimately provide new therapeutic strategies for a range of disorders.

## Figures and Tables

**Table 1:** Bacterial source, gene location, serotypes, receptors, and selective substrates for the enzymatic activity of botulinum neurotoxins. NA: Not available.

Group/ Bacteria	Toxin Serotypes/ Subtypes	Biochemistry	Substrate (Cleavage Site)	Substrate Location	Neurotoxin Gene Location	Botulism
*C. botulinum* group I	A1 to A10, A(B), Ab, Af, Af84, A2F4F5	Proteolytic	SNAP-25 (QR) SNAP 23 (AR)	Presynaptic plasma membrane	Chromosome or plasmid	Human/animal
	B1 to B3, B5 (Bc), B6, B7, Ba, Bf		VAMP (QF)	Synaptic Vesicle		
	F1 to F5		VAMP 1 (QK) and VAMP 2 (LE)	Synaptic Vesicle		
	X		VAMP 1, VAMP 2, VAMP 3, VAMP 4, VAMP 5 And Ykt6 (RA)	Synaptic Vesicle		
	H		VAMP 1, VAMP 2, VAMP 3 (LE)			
*C. botulinum* group II	B4	Non-proteolytic	VAMP 1 (QF)	Synaptic vesicle	Chromosome or plasmid	Human/animal
	E1, E2, E3, E6 to E10		SNAP25 (RI)	Presynaptic plasma membrane		
	F6		VAMP1, VAMP2, VAMP 3 (QK)	Synaptic vesicle		
*C. botulinum* group III	C, CD	Non-proteolytic	SNAP25 (RA), Syntaxin 1A (KA), Syntaxin 1B (KA), Syntaxin 2 (KA), Syntaxin 3 (KA)	Presynaptic plasma membrane	bacteriophage	Animal, very rare in human
	D, DC		VAMP1, VAMP2, VAMP 3 (KL)	Synaptic vesicle		
*C. botulinum* group IV (*C. argentinase*)	G	Proteolytic	VAMP 1, VAMP 2, VAMP 3 (AA)	Synaptic vesicle	Plasmid	No natural case reported
*C. butyricum* group V	F7	Non-proteolytic	VAMP 1, VAMP 2 (QK)	Synaptic vesicle	Transposon	Human
*C. baratii* group VI	E4, E5	Non-proteolytic	SNAP-25 (RI)	Pre-synaptic plasma membrane	Transposon	Human
Other organisms producing BoNTs	En (Enterococcus faecium strain)	Non-proteolytic	VAMP 2 (DL), SNAP25, SNAP 23 (KD), Syntaxin (MD)	Synaptic vesicle/Plasma membrane	Conjugative plasmid	NA
	BoNT/Wo or BoNT/I (Weissella Oryzae)	Non-proteolytic	VAMP2	Synaptic vesicle	NA	
	Cp1 toxin (BoNT homolog) *Chryseobacterium piperi*	?	?	?	?	

**Table 2:** Ganglioside-binding sites and receptors for different serotypes of botulinum toxin (for a detailed table, please refer to [3] (ND: Not determined).

Serotypes	Ganglioside	Ganglioside-Binding Site
BoNT/A	GT1b, GD1a, GD1b, GM1	H…SXWY…G
BoNT/B	GT1b, GD1a, GD1b, GM1	H…SXWY…G
BoNT/C	GD1b, GT1b, GD1a, GM1a	-ND-
BoNT/D	GD2, GT1b, GD1b, PE	DXY…VXN
BoNT/DC	GM1a, GD1a, GD1b, GT1b	-ND-
BoNT/E	GD1a, GQ1b, GT1b, GM1	K…SXWY..G
BoNT/F	GT1b, GD1a, GM3, GD1a, GM1	H…SXWY..G
BoNT/G	GT1b, GD1a, GD1b, GM3, GM1	G…SXWY..G
